# Consumption, the Most Dangerous Communicable Disease

**Published:** 1902-11

**Authors:** 


### Consumption, the Most Dangerous Communicable Disease.

---

At the meeting of the National Conference of Charities and Correction in Detroit, June 2, 1902, Dr. Baker, Secretary of the Michigan State Board of Health, said: "Not one of the common, so-called 'contagious' diseases is usually contracted by simple contact of the unbroken surface of a human body with the surface of an infected human body. Therefore the term 'contagious' implying as it does the spread of disease by contact, should be obsolete. A much better term is the single word, 'communicable.'

"Of all the communicable diseases consumption (pulmonary tuberculosis) is now the most dangerous. More people contract that disease than any other. Therefore anything, any statement, or any influence which belittles the importance of restricting the spread of consumption, does damage in the most vital point to the interests of the public health and safety.

"Improper housing and improper feeding of the poor are important evils to be done away with, because they lead to discomfort and lowered vitality, and tend to *spread* disease. But if the germs of tuberculosis were generally restricted, any amount of lowered vitality, because of improper housing and improper food, would not cause a single case of consumption.

"The essentials for the restriction of consumption are: first the general recognition of the truth that consumption is the most communicable disease. Knowledge of that fact is the power without which consumption cannot be restricted. It is lack of action because of ignorance of this great truth—that consumption is spread from infected persons—that kills off the improperly housed and improperly fed poor. It is ignorance of that great truth that kills off the rich by tubercular disease, in spite of proper housing and proper feeding.

"It is the slow but gradual gaining of that precious knowledge by the common people, and action governed by that knowledge, that is reducing the mortality from consumption, as it is being reduced in Michigan.

"In order to be most useful to the public, it is essential that this important knowledge shall be gained by, and shall govern the action of, every coughing consumptive, who otherwise is a constant source of danger. Therefore, the consumptive should be promptly put in possession of that knowledge. This first essential cannot be fulfilled by the public unless every case of well-developed consumption shall be reported to the health officer. Every case reported should be promptly informed how to avoid reinfection of the patient and spreading the disease."—*Public Health.*

